# Transcriptomic profiling of the human brain reveals that altered synaptic gene expression is associated with chronological aging

**DOI:** 10.1038/s41598-017-17322-0

**Published:** 2017-12-04

**Authors:** Allissa A. Dillman, Elisa Majounie, Jinhui Ding, J. Raphael Gibbs, Dena Hernandez, Sampath Arepalli, Bryan J. Traynor, Andrew B. Singleton, Dagmar Galter, Mark R. Cookson

**Affiliations:** 1 Cell Biology and Gene Expression Section, Laboratory of Neurogenetics, National Institute on Aging, National Institutes of Health, Bethesda, MD USA; 2 Molecular Genetics Section, Laboratory of Neurogenetics, National Institute on Aging, National Institutes of Health, Bethesda, MD USA; 3 Computational Biology Core, Laboratory of Neurogenetics, National Institute on Aging, National Institutes of Health, Bethesda, MD USA; 4 Genetic Technologies Group, Laboratory of Neurogenetics, National Institute on Aging, National Institutes of Health, Bethesda, MD USA; 5 Neuromuscular disease research Section, Laboratory of Neurogenetics, National Institute on Aging, National Institutes of Health, Bethesda, MD USA; 6 Department of Neuroscience, Karolinska Institutet, 171 77 Stockholm, Sweden

## Abstract

Aging is a biologically universal event, and yet the key events that drive aging are still poorly understood. One approach to generate new hypotheses about aging is to use unbiased methods to look at change across lifespan. Here, we have examined gene expression in the human dorsolateral frontal cortex using RNA- Seq to populate a whole gene co-expression network analysis. We show that modules of co-expressed genes enriched for those encoding synaptic proteins are liable to change with age. We extensively validate these age-dependent changes in gene expression across several datasets including the publically available GTEx resource which demonstrated that gene expression associations with aging vary between brain regions. We also estimated the extent to which changes in cellular composition account for age associations and find that there are independent signals for cellularity and aging. Overall, these results demonstrate that there are robust age-related alterations in gene expression in the human brain and that genes encoding for neuronal synaptic function may be particularly sensitive to the aging process.

## Introduction

Aging is an inherent, multidimensional biological process that includes aspects of physical and behavioral changes. Despite being universal in nature, and representing a major risk factor in multiple degenerative conditions, defining the biological basis of aging remains challenging. One general approach to understanding the basis of aging has been to examine correlative measures of chronological age. Because of the postulated role of genetics in aging^1,2^, gene expression has been measured in many tissues and species, including brain^3–5^. Similarly, epigenetic measures of aging have been shown to be reliable across multiple tissues^6^. We have previously reported on age-associated changes in gene expression^7^ and CpG methylation^8^ in the human brain. Collectively, these studies demonstrate that there are multiple epigenetic and gene expression changes that are associated with aging.

There have been many previous large-scale studies of gene expression in aging using microarrays^9–14^. However, the development of RNA-sequencing (RNA-Seq) has shown that microarrays have several limitations including a narrow dynamic range that can lead to underestimation of differential gene expression^15^. Recent studies have indicated that RNA-Seq can be used to reliably estimate gene expression in the brain, for example to show changes that occur with development in rodents^16^ and humans^17^.

Here, we perform an RNA-Seq analysis on the dorsolateral prefrontal cortex, an anatomically well-defined region of the brain available from many brain banks and used in other transcriptomic studies^18–20^, from a series of individuals across human aging. Using weighted gene correlation network analysis (WGCNA) to identify groups of genes with similar expression patterns^21^, as previously applied to several microarray studies of the brain^9–14^, we find evidence of many relationships between gene expression and brain aging. We extensively validate these observations against publically available datasets and address brain regional effects and estimate the likely contribution of changes in cellular composition to gene expression. Collectively, the results provide a series of newly identified changes in human brain aging with a particularly strong regional association with loss of synaptic gene expression.

## Results

### Detection of modules of co-expression in the human brain

Using samples from the frontal lobe of the cerebral cortex of 56 human subjects from 15–79 years of age (Supplementary Fig. S1 and Supplementary file 1), we generated RNA-Seq expression data with a mean of 242,340,441 bp paired end reads (range 85,325,632–548,398,101). From these, we could align an average of 96.5% of reads (range 89.1–98.2%) to the human genome (Supplementary file 2). The final dataset has 39,460 transcripts, mapped to 11,648 genes, that were expressed more than one SD from the mean of intronic expression and detected in >95% of subjects.

**Figure 1:**
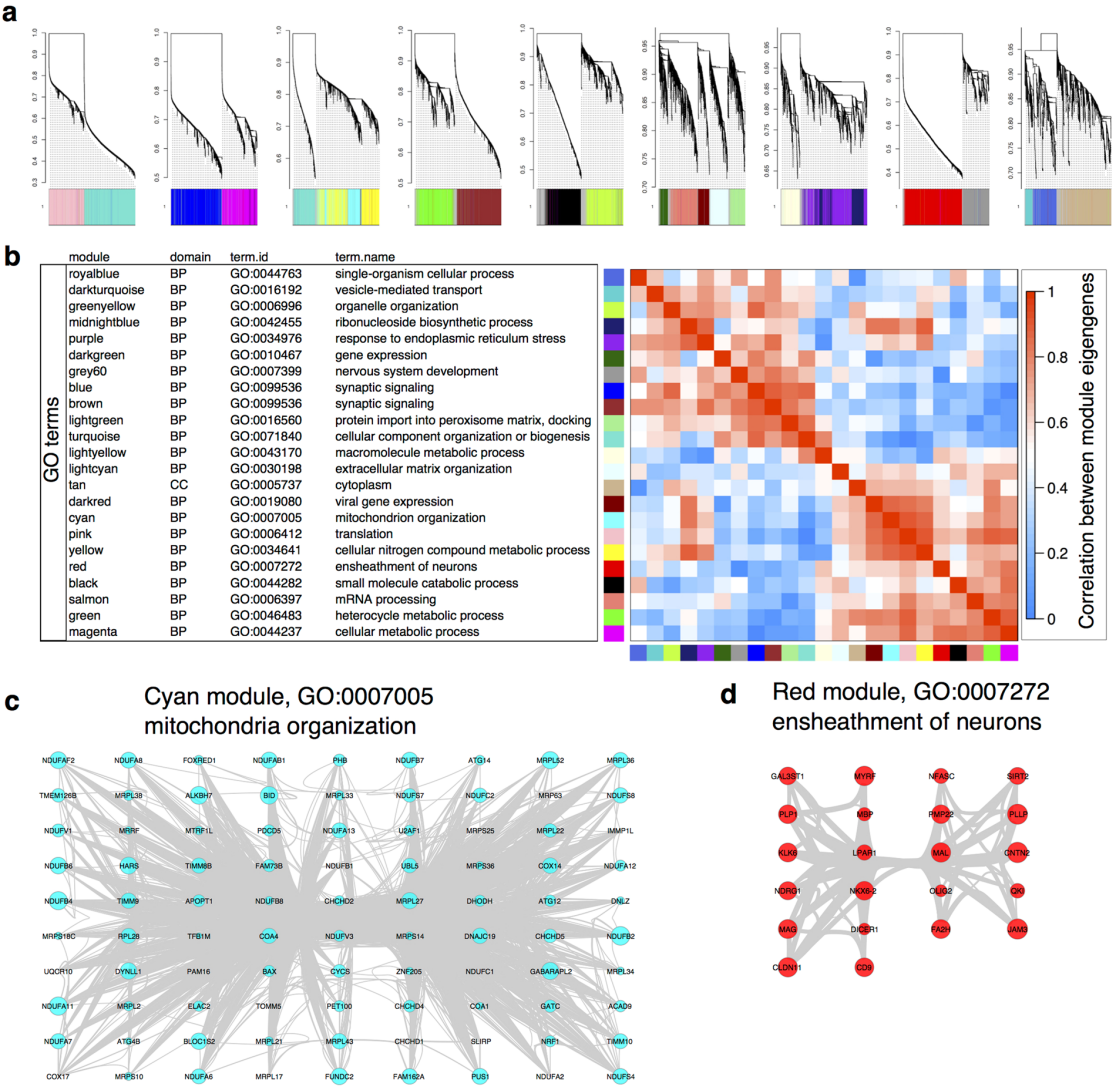
WGCNA derived modules of gene expression. (**a**) An overall view of the network of gene expression. Height on the y-axes represents the dissimilarity of detected transcripts from the overall topology of gene expression. Colors below each line show the modules to which each transcript was assigned. (**b**) The eigengenes for each module are enriched for specific biological processes. For each module, we performed gene ontology (GO) enrichment, listing the term.id and name for each module (see also supplementary file 4 for p values). The module eigengenes showed correlation with each other, as indicated in the heatmap on the right. (**c**,**d**) Examples of modules enriched for biological terms. We used cytoscape to visualize organization of genes assigned to the GO category mitochondria organization in the cyan module (**c**) or the GO category ‘ensheathment of neurons’ in the red module (**d**). Circles are sized by the within module connectivity (Kwithin).

We used WGCNA to examine transcripts that were co-expressed within the data. The network approximated a scale-free topology at a soft thresholding power of 6 (Supplementary Fig. S2) and contained 37,029 transcripts assigned to 23 discrete modules, with 2611 transcripts not assigned to a specific module (Fig. 1a and Supplementary file 3).

To classify the WGCNA modules, we first performed enrichment of gene ontology (GO) terms (Supplementary file 4), which provided unique categorizations for 22 modules in the domain of biological processes (summarized in Table 1). We also identified the most highly connected hub transcripts within each module (Table 1). The WGCNA modules reflected both functional and cellular processes and, at least in some cases, there was an obvious biological connection between the hub genes and the GO:enrichment for biological processes. For example, the cyan module was highly enriched for GO: 0007005 (Fig. 1c), representing mitochondrion organization, and the most highly connected hub gene, NDUFA2, encodes the mitochondrial NADH:Ubiquinone Oxidoreductase Subunit A2 (Table 1). Some modules appeared to represent specific cell types, including the red module that was enriched for GO: 0007272, ensheathment of neurons that, in turn, contained multiple genes associated with myelinating cells such as MBP, MAG and PMP22 (Fig. 1d).

**Table 1:** Summary of WGCNA analysis.

module	GO enrichment				Module hub			
	term.id	term.name	p.value	# genes	transcript	gene	kTotal	kWithin
black	GO:0044282	small molecule catabolic process	7.34E-19	54	uc001ffr.3	PBXIP1	1250	627
blue	GO:0099536	synaptic signaling	1.89E-17	95	uc010abi.3	NBEA	2525	900
brown	GO:0099536	synaptic signaling	5.70E-19	90	uc001bto.3	BAI2	2124	779
cyan	GO:0007005	mitochondrion organization	1.14E-33	95	uc021yem.1	NDUFA2	1434	327
darkgreen	GO:0010467	gene expression	3.22E-09	109	uc003xkt.5	ASH2L	906	67
darkred	GO:0019080	viral gene expression	2.06E-25	32	uc003hza.3	RPL34	1473	164
darkturquoise	GO:0016192	vesicle-mediated transport	3.69E-05	23	uc031pyt.1	APBB1	1180	55
green	GO:0046483	heterocycle metabolic process	4.52E-10	284	uc010wqb.2	CEP95	1766	364
greenyellow	GO:0006996	organelle organization	1.28E-08	184	uc011lhl.2	ATP6V1C1	1522	279
grey	GO:0034641	cellular nitrogen compound metabolic process	2.59E-11	482	uc003kxw.3	SOWAHA	1160	154
grey60	GO:0007399	nervous system development	5.35E-09	69	uc003nqg.3	HLA-E	586	189
lightcyan	GO:0030198	extracellular matrix organization	3.76E-16	33	uc001nsa.3	DAGLA	2339	137
lightgreen	GO:0016560	protein import into peroxisome matrix, docking	0.00963	3	uc010kgt.2	MAP7	838	150
lightyellow	GO:0043170	macromolecule metabolic process	4.66E-08	236	uc010yyo.2	DBI	1880	549
magenta	GO:0044237	cellular metabolic process	1.27E-11	467	uc009znf.2	SMUG1	1247	211
midnightblue	GO:0042455	ribonucleoside biosynthetic process	0.000362	12	uc001bbe.1	AX747516	2166	585
pink	GO:0006412	translation	3.08E-32	105	uc003ovj.2	LRRC73	1452	315
purple	GO:0034976	response to endoplasmic reticulum stress	0.0196	25	uc010sqb.2	ERBB3	1551	855
red	GO:0007272	ensheathment of neurons	2.57E-11	22	uc003bew.2	PRR5	778	108
royalblue	GO:0044763	single-organism cellular process	1.94E-06	189	uc022cbu.1	TCEAL3	1168	239
salmon	GO:0006397	mRNA processing	2.76E-28	71	uc002nxp.3	SCN1B	771	206
tan	GO:0005737	cytoplasm	6.47E-06	334	uc010jie.1	LARP1	2605	1785
turquoise	GO:0071840	cellular component organization or biogenesis	3.80E-33	856	uc002bmi.1	MRPL46	1798	470
yellow	GO:0034641	cellular nitrogen compound metabolic process	4.78E-13	399	uc001ffr.3	PBXIP1	1250	627

A table showing the WGNCA modules with the most significant enrichments for GO terms, along with associated number of genes in the module that were enriched for that GO term and the associated p value for enrichment.

### Many transcript level associations between gene expression and aging

We next examined the correlations of eigengenes for each module with both technical (RNA integrity number [RIN], post-mortem interval [PMI] and batch in which the sample was sequenced) and biological (gender and age) variables (Supplementary Fig. S3). There were only moderate associations with technical variables. For example, the strongest association for RIN was with the darkgreen module (R = 0.36, adjusted p = 0.007). There were no significant associations between module eigengene expression and gender, which may be due to the relatively small number of male brains in our final dataset (14 from 56 samples). In contrast, there were 6 module eigengenes that showed significant positive and 8 that showed significant negative associations with age (Supplementary Fig. S3). These analyses suggest that age strongly correlates with gene expression in the brain. We therefore looked in turn at the most significant modules that were positively and negatively associated with aging in brain.

**Figure 2:**
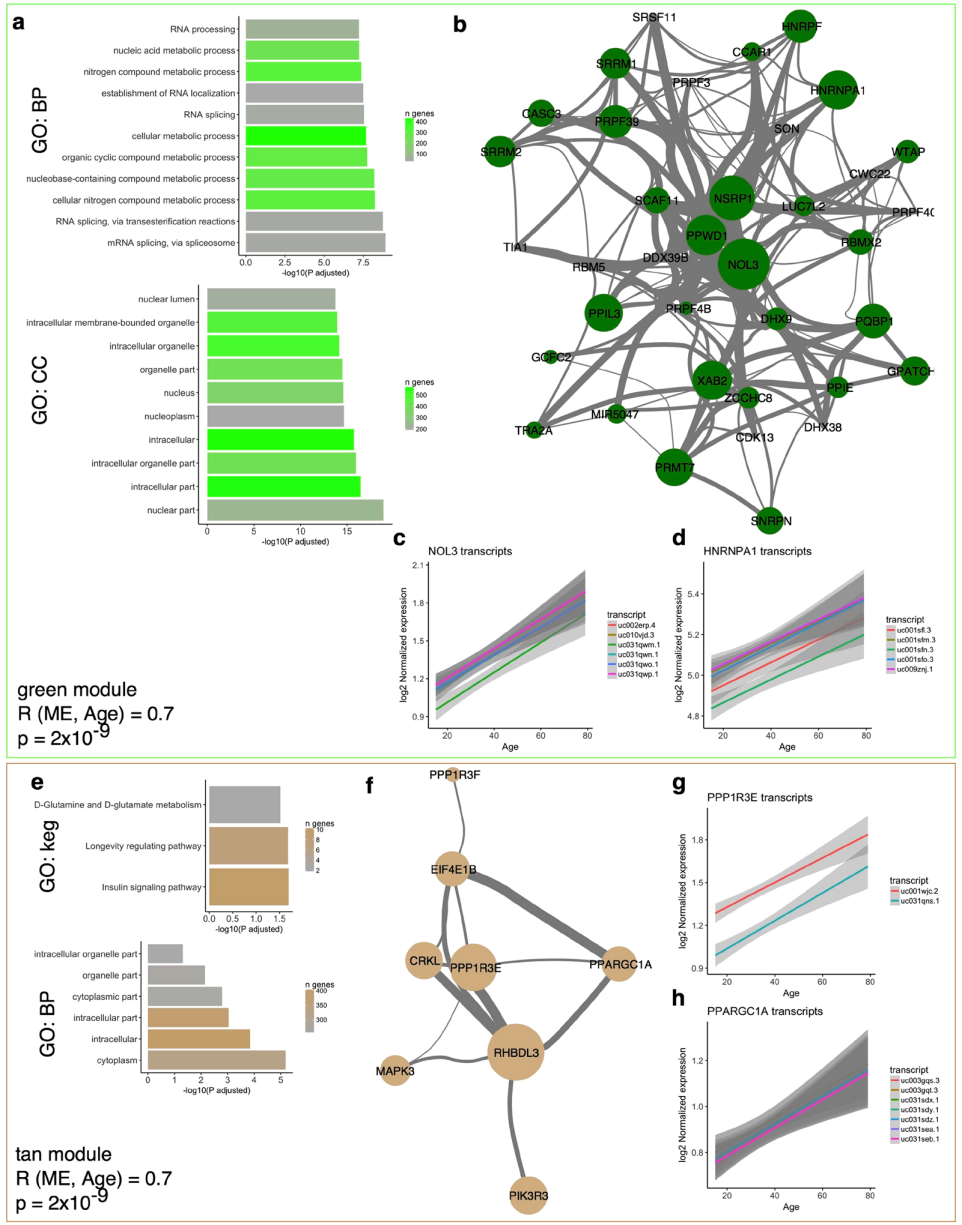
Genes that accumulate with aging in the brain include RNA metabolism and longevity genes. (**a**) The green module, with a correlation between module eigengene and age of 0.7 (p = 2 × 10^−9^), contained multiple transcripts enriched for GO terms related to RNA splicing (upper panel) and the nucleus (lower panel). Horizontal axes show the −log10 of the p value for enrichment for the listed terms on the vertical axis and bars are shaded by the number of genes overlapping between the green module and the number of genes in the GO term. (**b**) Visualization of the relationship of genes in the category GO:0000377 ‘RNA splicing, via trans-esterification reactions with bulged adenosine as nucleophile’ in the green module. Nodes are sized by the correlation with age for the most highly expressed transcript per given gene and linewidths indicate the strength of correlation between genes within the WGCNA analysis. (**c**,**d**) Individual associations with age for multiple transcripts for the genes NOL3 (**c**) and HNRNPA1 (**d**) with each transcript given a different color. Shaded regions indicate the 95% confidence intervals for the regression for each transcript. (**e**) The tan module (R for age 0.7, p = 2 × 10^−9^) showed enrichment for insulin signaling and longevity regulating genes. (**f**) Visualization of the kegg:04910 ‘Insulin signaling pathway’ within the tan module, with the addition of the highly associated gene RHBDL3. Nodes are sized by the correlation with age for the most highly expressed transcript per given gene and line widths indicate the strength of correlation between genes within the WGCNA analysis. (**g**,**h**) Individual associations with age for multiple transcripts for the genes PPP1R3E (**g**) and PPARGC1A (**h**), with each transcript given a different color. Shaded regions indicate the 95% confidence intervals for the regression for each transcript.

The eigengene for the green module (2081 transcripts from 777 genes) showed a strong positive correlation with age (R = 0.7, adjusted p = 2 × 10^−9^). GO profiling demonstrated multiple categorizations related to RNA metabolism and the nucleus (Fig. 2a). Specific genes in this module included NOL3 that encodes a nucleolar protein (Fig. 2b,c; R = 0.67, p = 1.3 × 10^−8^ for the association of transcript uc031qwm.1 with age) and heteronuclear ribonucleoproteins including HNRNPA1 (Fig. 2b,d; R = 0.53, p = 2.3 × 10^−5^ for uc001sfn.3). These associations of nuclear genes with aging may indicate active control of gene expression during the aging process (see discussion). There was an equally strong positive association with age for the eigengene of the tan module (R = 0.7, p = 2 × 10^−9^). The strongest individual association in this module was with RHBDL3, a rhomboid-like protease that we have previously nominated^7^ as associated with age (R = 0.809, p = 2.56 × 10^−14^ for transcript uc010csx.1). This module was not associated with a unique GO enrichment for biological process, but was enriched for the kegg pathways, insulin signaling pathway and longevity regulating pathways. The tan module included the genes protein phosphatase PPP1R3E (Fig. 2g; R = 0.65, p = 7.23 × 10^−8^ for uc001wjc.2) and PPARGC1A (Fig. 2h; R = 0.47, p = 0.0003 for uc031sdy.1), the latter encoding the transcriptional coactivator PGC1α. Prior evidence has linked aging to specific genes such as PGC1α^22^ but also the more general process of insulin signaling^23^, suggesting the associations of gene expression with age in this sample series have biological validity.

**Figure 3:**
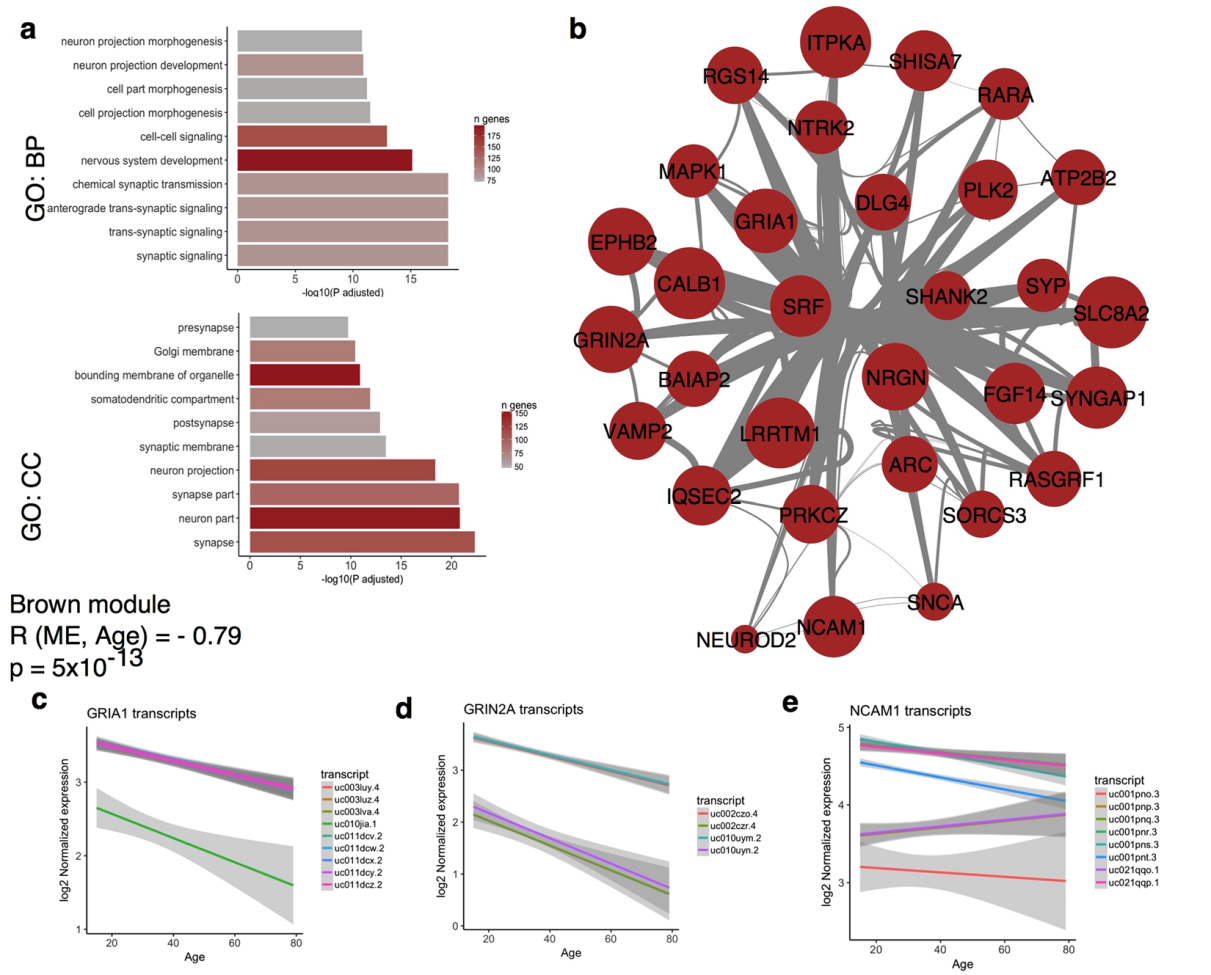
Diminished expression of transcripts encoding synaptic proteins with age. (**a**) GO enrichment for the brown module shows multiple strong enrichments with aspects of neuronal function, with the −log(P) for each GO term shown on the x-axis for multiple terms on the y-axis. Bars are shaded by the number of genes from the blue module that overlap with the GO term for each category. (**b**) Visualization of the GO:0048167 term ‘regulation of synaptic plasticity’ within the brown module. Nodes are sized by the inverse of the correlation with age for the most highly expressed transcript per given gene and line widths indicate the strength of correlation between genes within the WGCNA analysis. (**c–e**) Individual associations with age for multiple transcripts for the genes GRIA1 (**c**), GRIN2A (**c**) and NCAM1 (**d**), with each transcript given a different color. Shaded regions indicate the 95% confidence intervals for the regression for each transcript.

Conversely, there was a strong negative association with age for the brown module eigengene (R = −0.79, adjusted p = 5 × 10^−13^). In this module, we found a strong accumulation of genes encoding synaptic proteins (Supplementary file 4), with the GO term synaptic signaling significantly enriched (adjusted p = 5.7 × 10^−19^; Table 1). Several transcripts from genes coding for synaptic proteins including both AMPA (Fig. 3c; gene GRIA1; R = −0.696, p = 2.6 × 10^−9^ for uc011dcy.2), NMDA (Fig. 3d; GRIN2A: R = −0.728, p = 2.08 × 10^−10^ for uc010uym.2) and Kainate type glutamate receptors showed negative associations with age. However, changes in neurotransmitter genes were not limited to excitatory glutamate systems, as we also found decreased expression of inhibitory GABA subunits with age (Supplementary Fig. S4h,i). Our data further suggest that as well as there being changes in overall expression of many genes with aging, some genes may additionally show splicing alterations. For example, while some transcripts for the neuronal cell adhesion molecule NCAM1 showed negative associations with age (Fig. 3e; R = −0.656, p = 4.12 × 10^−8^ for uc001pns.3), other transcripts for the same gene showed no significant associations with age (Fig. 3e; uc021qqo.1, R = 0.181, p = 0.2).

Outside of neuronal genes such as neurotransmitters and adhesion molecules, some more general cellular markers also showed negative associations with age. These include the royal blue module (R for module eigengene and age −0.74, p = 5 × 10^−11^) that contains the inflammatory mediator TGFB1 (Supplementary Fig. S4a–c) and the purple module (R = −0.59, p = 2 × 10^−6^ for module eigengene and age), which was classified with the GO term response to endoplasmic reticulum stress and included transcripts for the known ER stress mediator ATF6 (Supplementary Fig. S4d–f). As genes such as TGFB1 and ATF6 are expressed in non-neuronal cells, these data show that not all genes associated with age are in neuronal modules.

### Validation and estimation of brain regional effects

To determine if the associations of genes with aging were robust, we first compared the associations with aging to previously generated microarray datasets from our lab using the same subjects^7^. To provide equivalency with prior data, we removed genes that showed divergence in significance between transcripts mapped to the same gene, selected the highest expressed single transcripts in the RNAseq data and matched to all genes also detected in the array dataset (see methods). We found a positive correlation (R = 0.711, p < 10^−16^) between association of age and each gene in the RNA-Seq and array datasets (Supplementary Fig. S5a). We further validated these results using an independently ascertained sample series^24^, where again there was a positive correlation between the effects of aging with our RNA-Seq data (R = 0.483, p < 10^−16^_;_ Supplementary Fig. S5b). The similarity between the current dataset and previously microarray datasets suggests that the age associations nominated here are platform-independent and therefore robust, noting that this considers only genes estimated in both RNA-Seq and arrays and therefore does not consider genes only found in the former dataset.

**Figure 4:**
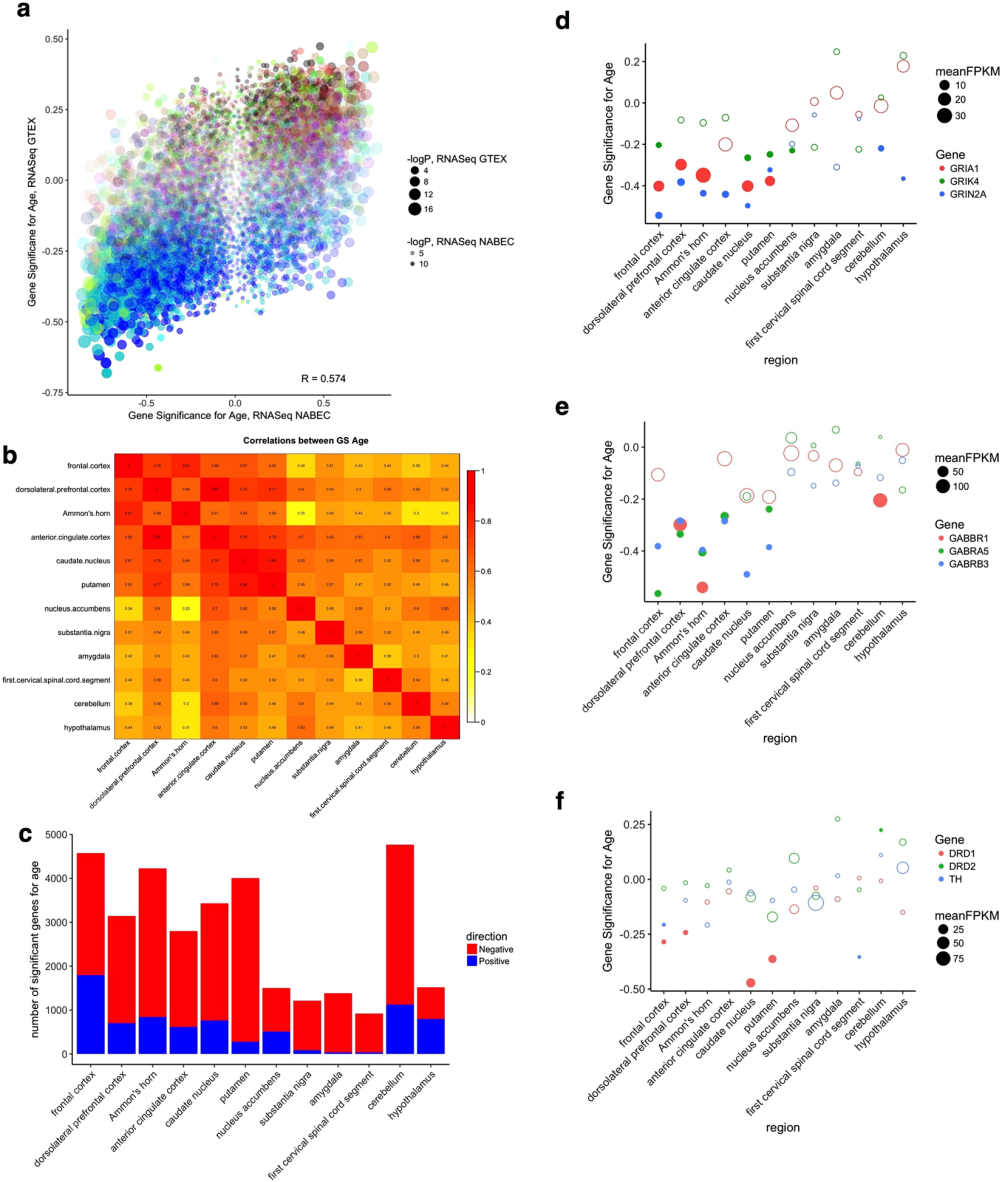
Validation of age associations against GTEx data and regional events (**a**) Overall consistency between the correlations of gene expression and age in our dataset (x axis) and the GTEx frontal cortex (y axis). Each point represents a different gene and is colored by the WGCNA module in our data and sized by −log10 of the p value for association with age, (**b**) Correlations between age associations in different brain regions in the GTEx dataset. For each pair of brain regions, the overall correlations between age associations are colored by strength; actual values of R are given in each box. (**c**) Counts of numbers of genes showing significant (nominal p < 0.05) associations with age in each brain region along the horizontal axis, with negative associations in red and positive associations in blue. (**d–f**) Specific genes showing significant associations with aging from our dataset tested in the GTEx brain regions, specifically excitatory glutamate receptor (**d**), inhibitory GABA genes (**e**) and genes related to dopamine neurotransmission (**f**). The vertical axis shows the correlation with age for each of three genes in different colors for different brain regions in the GTEx dataset along the horizontal axis. Points are sized to the mean expression (as FPKM) in each brain region and filled circles indicate significant (nominal p < 0.05) associations with age.

To further validate the associations of gene expression using an RNA-Seq dataset, we used the publically available GTEx dataset that contains independently ascertained samples. As the GTEx data is summarized to the gene, not transcript level, we again picked the highest expressed transcript for each gene in our data to provide an equivalent comparison. As for the observations with previous microarrays, there was a positive correlation (R = 0.574, p < 10^−16^) in the estimates of association with age for all genes (Fig. 4a).

Collectively, these results support the hypothesis that there are many age- associations with gene expression in the human brain that are robust to sample series and gene expression platform.

An important advantage of the GTEx dataset over other sample series is that multiple brain regions were sampled. Therefore, we additionally compared age associations across all brain regions. In general, the brain areas that are anatomically and evolutionarily related showed greater consistency in the associations between aging and gene expression (Fig. 4b and Supplementary Fig. S6). For example, the overall correlation for age-related associations between the frontal cerebral cortex and dorsolateral prefrontal cortex was 0.76 when considering only genes expressed in both regions. The same measure between two areas of the striatum, the caudate and putamen was 0.84. In contrast, more distant areas such as the spinal cord and frontal cortex showed much lower correlations (R = 0.44).

Prior studies have suggested that there are regional differences in the associations of aging with gene expression and that more recently evolved structures show enhanced associations with aging. To test this, we counted the number of significant associations in the GTEx dataset (Fig. 4c). The frontal cortex showed the highest number of associations, with other brain regions generally showing fewer associations in a similar order as the overall correlations between regions, except for the cerebellum that showed the second highest number of associations after the frontal cortex.

Finally, to examine associations with specific genes, we looked at excitatory (Fig. 4d) or inhibitory (Fig. 4e) neurotransmitter receptor candidates from our own dataset in the different GTEx brain regions. We confirmed that there are significant age associations with five genes (GABRA5, GABRB3, GRIK4, GRIN2A and GRIA1), all in the same direction and a similar magnitude to our dataset in the GTEx frontal cortex data. From the validated genes, we found substantial variation across regions. For example, GRIN2A showed significant negative association with age in all brain regions except amygdala, spinal cord, nucleus accumbens and substantia nigra, despite being adequately detected in all brain regions (Fig. 4d). Finally, we looked at genes related to dopamine neurotransmission that showed more variable expression across brain regions, specifically tyrosine hydroxylase (TH) and two major dopamine receptor genes, DRD1 and DRD2 and noted that the latter two genes showed a significant correlation with age in the caudate and putamen, two major regions of dopaminergic innervation from the substantia nigra (Fig. 4f). These results show that different brain regions have differential responses to aging.

### Contribution of cellularity changes with aging

**Figure 5:**
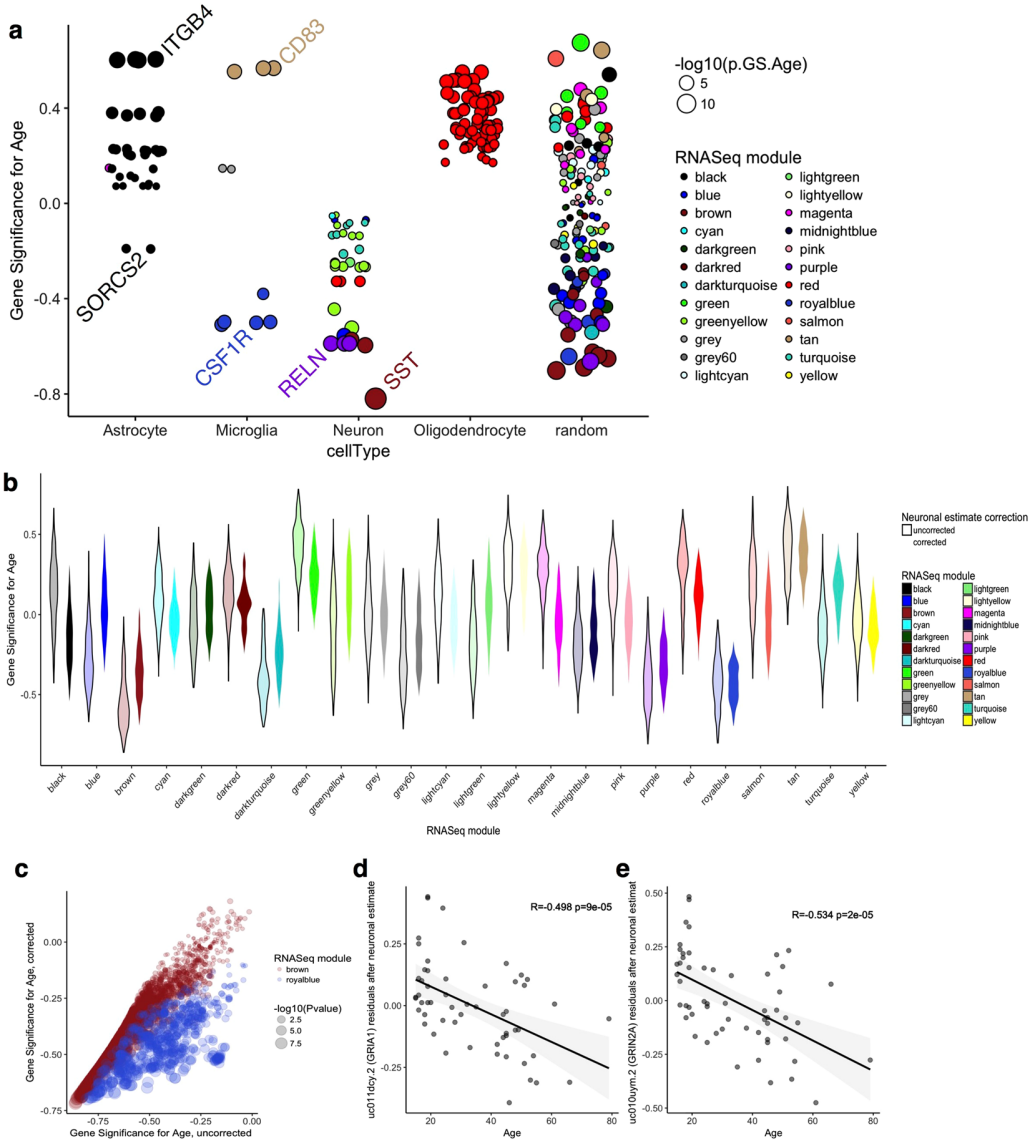
Population-specific expression analysis disambiguates age-related changes in cellularity from cell-dependent gene expression. (**a**) Correlation (GS) between age and transcript expression for genes representative of the major classes of cells in the CNS (horizontal axis) or a similar number of randomly selected genes. Genes are sized by the −log10 of the p value for association with age and colored by the WGCNA module assignments in our RNA-Seq dataset; note that all available transcripts were used for each gene. (**b**) Violin plot of the distribution of values of R with age (vertical axis) for all transcripts in a given WGCNA module (horizontal axis) either without (lighter colors outlined in black) or after correction for the proportion of neuronal cells in each sample (darker colors). (**c**) Comparison of uncorrected (horizontal axis) and neuron-corrected (vertical axis) associations with age for all transcripts in the brown, synaptic, module, that showed an overall negative correlation with age. Points are sized to the p value for association with age in the corrected samples. (**d**,**e**) Associations with age remain for specific transcripts after correction for neuronal proportions. The residuals after linear regression for neuronal markers are plotted on the vertical axis for each sample of different ages on the horizontal axis for transcript uc011dcy.2 (GRIA1, **d**) and uc010uym.2 (GRIN2A, **e**).

We considered that these effects on synaptic gene expression might be related to changes in the cellular composition of the samples with aging. Specifically, it has been suggested that there is a loss of subtypes of neurons in the dorsolateral prefrontal cortex with age in different species^25,26^. We have also recently shown, using microarray data, that there are potential changes in glial cell gene expression with aging that are consistent across brain regions^27^. Therefore, loss of synaptic gene expression with aging might feasibly be accounted for by changes in the proportion of neuronal and glial cells with age. To address this potential confounding variable, we first examined expression of genes representative of the major populations of neurons and glial cells in each population, we used genes identified as differentially expressed between cell types in a recent single cell analysis of human brain^28^ and confirmed that these are authentic markers represented specific cell types by cross-referencing with additional RNA-Seq data (see Methods). Plotting out the associations with age in this set of genes (Fig. 5a) showed that neuronal genes tended to have negative associations with age whereas marker genes of oligodendrocytes tended to increase with age. An identical pattern was seen in the GTEx frontal cortex data although the decrement in neuronal restricted genes was less pronounced in GTEx cerebellum data (Supplementary Fig. S7).

To further discriminate between the effects of loss of neurons compared to loss of neuronal gene expression, we used population specific expression analysis (PSEA^29,30^) to estimate and correct for proportions of neuronal and glial cells in our RNA-Seq dataset. Using the same set of cell-specific marker genes as above, we constructed a marker reference for each cell type for each sample. These markers had the expected property of correlating well with known marker genes for each cell type, and were distinct from those genes used to generate the marker (Supplementary Fig. S8a). Plotting age against proportion of cells further confirmed loss of overall neuronal gene expression and increases in oligodendrocyte markers with age (Supplementary Fig. S8b).

We noted that many of the top associations were not in cell type restricted genes (Supplementary Fig. S8c). For example, TMEM198 (R = −0.86; p = 1.7 × 10^−7^ for association with age in WGCNA) showed association with markers of neurons, but also with astrocytes and microglia (Supplementary Fig. S8c). Conversely, not all neuronal restricted genes showed negative associations with age. For example, both SST and TMEM130 are both restricted to neurons and correlated with neuronal estimates from PSEA but only SST showed a negative association with age (Supplementary Fig. S9). These data suggest that the expression of some, but not all, neuronal genes might be influenced by cellularity.

To further test the contribution of cellularity to altered gene expression with aging, we generated residuals of gene expression from a linear model using the neuronal population marker, thereby correcting the raw expression data for the number of neurons in each sample. Comparing the relationship of associations of gene expression with age in each WGCNA module in uncorrected and corrected analyses demonstrated that loss of neurons accounts for some, but not all, of the observed associations, as demonstrated by the decrease in estimate of association with age across the modules (Fig. 5b). For example, within the brown ‘synaptic’ module that showed strong negative associations with age, the number of significant associations decreased from 2356 to 1170 after correction for neuronal markers (Fig. 5c). Some modules were more robust to correction for neuronal estimate, including the royal blue module that contained several transcripts for the microglial gene Colony stimulating factor 1 receptor (Fig. 5a,b). Importantly, even after correction for neuronal estimates, many significant associated remained, including GRIA1 and GRIN2A (Fig. 5d,e). Similar patterns of variable correction for neuronal estimates were seen in the GTEx dataset (Supplementary Fig. S10). Collectively, these results show that while some of the gene expression signal associated with age may be related to differences in cellularity, there are also likely associations independent of cell type.

## Discussion

Identifying age-related changes in gene expression is one of several ways in which the aging process can be defined. Here we examined gene expression in the brain using RNA-Seq and a sample series that we previously have examined with microarrays^7,27^ as a way to explore the biology of human aging.

Consistent with prior studies using microarrays, gene expression in the brain appears to be robustly organized into modules to which WGCNA is sensitive^31–37^. These modules of gene expression reflect organization of brain structures and function, being readily categorized by gene ontology related to specific cell types or subcellular organelles, the latter including mitochondria and synaptic vesicles.

Our dataset identified a series of associations of gene expression with aging, including several that have been proposed previously based on microarrays^7,27^. A striking novel finding was the robust negative associations of genes encoding synaptic proteins with age. These expression changes are likely related to functional changes in synaptic integrity seen with aging in a variety of contexts^38–40^. Because our RNA-Seq dataset contains a relatively large number of detected genes, we could show that synaptic changes occur across multiple neurotransmitter types, including both excitatory glutamateric and inhibitory GABAergic synapses. Furthermore, genes encoding both pre- and post-synaptic proteins are affected, potentially in tandem. Confirming these in the GTEx dataset allowed us to demonstrate regional variation in synaptic protein expression, with some of the strongest effects in areas of the cortex, the hippocampus (Ammon’s horn) and striatum. It is of interest that these areas have undergone relatively recent evolutionary changes in humans, including the expansion and involution of the cerebral cortex^41^. However, not all transmitter types are affected in all brain regions as, for example, D1 type dopamine receptors are resistant to aging effects and D2 type receptors decline mainly in the two regions with highest expression of these genes, the caudate and putamen.

Given the apparent strength and validity of changes in synaptic gene expression in this study, it is important to consider why these have not been widely reported in prior array studies, including our own studies using the same source material^7^. Examining prior data, we note that strong candidates such as GRIA1 or GRIN2A were not reliably assayed in our previous microarray study and therefore were not able to be considered previously. Furthermore, the overall distribution of values of association with age was broader in RNASeq (Supplementary Fig. S11), assisting in detection of age-assocations.

A general concern with estimates of gene expression in the brain is that they are influenced by heterogeneous cellularity. For example, prior studies have reported activation of microglia and astrocytes with aging^27,42^ and, as noted above, there is evidence of neuronal loss in the brain region used to generate our dataset^25,26^. However, our data suggests that such changes in cellularity do not fully explain overall gene expression associations with age based on several independent considerations. First, some single gene markers of neurons do not show changes with age while others show relatively large changes with some intermediates. Second, the deconvolution of gene expression signatures with PSEA demonstrated a residual association with age. Third, although there is imaging-based evidence of loss of volume in some regions with aging, the estimated rates of loss are in the order of 0.2–0.5% per year^43,44^. For a strongly associated transcript such as GRIA1, there was a ~50% decrease in expression over the range of our dataset (64 years). Nonetheless, it is hard to formally reject the possibility that there are neurons, and potentially even subsets of neurons, that are lost with aging. Given that PSEA-based correction for neuronal estimates lead to a net decrease in the number of significant associations with age, it is likely that the age associations seen in this study are a mixture of changes in cellularity and gene expression changes per cell. It would be of interest in the future to look at age-related associations in gene expression at the single cell level. We also note that we have not confirmed any gene expression associations at the protein level, which would be an important future extension of the current dataset.

A difference between prior studies and the current approach is that we estimated transcript level associations rather than aggregating to the gene level. In line with expectations, multiple transcripts from the same gene generally correlated with each other, presumably due to expression at the overall gene level. However, there were examples for specific genes (e.g., NCAM1) where some transcripts show age association while others do not, which presumably reflect alternate exon usage driven by splicing changes with aging. The current study, with a limited number of subjects, is unlikely to be adequately powered to explore the full associations of splicing with aging in the brain, but future experiments could be designed with more samples and greater read depth to assess if there generalized associations between splicing and aging in the brain.

Overall, these results show that RNA-Seq provides an insight into the process of brain aging and that, in humans, robust changes in synaptic proteins at transcript levels can be detected in the aging brain. We speculate that these associations may be relevant to the study of age-related neurological and psychiatric disorders, as well as some of the well documented changes in brain function with normal aging.

## Methods

### Ethical Approval

Experiments using human *post-mortem* materials were approved by the National Institutes of Health Office of Human Subjects Research (OHSR) after determination to be Not Human Subjects research due to the de-identified nature of the samples. All procedures were performed in accordance with guidelines of the National Institutes of Health.

### RNA extraction and sequencing

Total RNA was extracted from human frontal cortex using a glass dounce tissue homogenizer and trizol. An Agilent 2100 Bioanalyzer RNA Nano Chip was utilized to measure the RNA quality; samples had a mean RIN of 7.9 (+/−1.1). Poly(A) + RNA was purified from 10ug of total RNA using oligo-dT magnetic beads. cDNA libraries were synthesized using the mRNA preparation kit (Illumina cat. no. RS-100-0801) as per the manufacturer’s protocol (http://mmjggl.caltech.edu/sequencing/mRNA-Seq_SamplePrep_1004898_D.pdf). A single library was run on each flowcell lane of an Illumina Hi-Seq sequencer generating 100 bp paired end reads. Sequence alignments.

We used the high-performance computational capabilities of the Biowulf Linux cluster at the National Institutes of Health, Bethesda MD (http://biowulf.nih.gov). Fastq files were aligned to the human reference genome (hg19) using GSNAP^45^. Transcript estimates were quantified with RSeQC^46^ and normalized using CQN^47^. Overall quality and total read counts are listed in Supplementary File 1.

Whole genome co-expression Network Analysis (WGCNA) Normalized transcript expression was used as an input for the R package WGCNA^21^. Gene expression modules within a signed network were generated in a blockwise fashion using a soft-thresholding power of 6, which approximated a scale-topology (Supplementary Fig. S2). The minimum size of the modules was set at 200 and cut height at 1. The eigengenes for each module were correlated against RIN of the sample, PMI, batch in which the sample was sequenced, gender and age of the donor. Modules were classified for gene ontology enrichments using gProfileR^48^ within R. We used cytoscape for module visualization, with the most highly expressed transcript in the dataset for each gene used to represent that gene. Either grid layout or the yFiles organic layout were used for subsets of genes identified by specific GO terms within a modules and edges were bundled using default settings in cytoscape.

### Validation against other microarray and RNA-Seq datasets

For validation, we used three independently ascertained sample series; our own sample series with gene expression estimated using Illumina beadchip arrays^7^; the frontal cortex series from Colantuoni *et al*., assayed using a custom array^24^; and the GTEx dataset that used RNA-Seq but with multiple brain regions and estimated gene expression per gene rather than per transcript. In each case, we used the dataset provided normalized values (e.g. FPKM reads for GTEx) to calculate the association with age (and nominal p value) and gene expression for each probe using the WGCNA package. We then matched genes and compared R values in each series. Where there were multiple potential matches, for example where we had multiple transcripts in our dataset to compare against gene-level estimates in GTEx, we used the transcript with the highest mean expression in our dataset. We did not consider genes in our dataset where multiple transcripts diverged widely, or genes in the array datasets that were not detected.

### Population specific expression analysis (PSEA)

We used PSEA essentially as described previously^29,30^. To construct sets of reference marker genes for the principal cell types of the CNS, we used the most highly differentially expressed genes identified by single cell RNA-Seq.^28^ and manually removed any genes that showed ambiguous cell type specificity based on available data at http://web.stanford.edu/group/barres_lab/brainseqMariko/brainseq.2.html that incorporates mouse brain single cell RNA-Seq.^49^. We then used multiple regression using theses sets of markers to estimate the proportions of neurons, astrocytes, oligodendrocytes and microglia in each sample. Conversely, we used the residuals after linear regression for neuronal marker estimate per sample for each probe to correct age associations for number of neurons.

### Data availability

Raw gene expression data has been submitted to the database of Genotypes and Phenotypes (dbGaP) with the accession number **phs001353.v1.p1**, entitled NABEC mRNA Sequencing of Human Cerebral Frontal Cortex.

## Electronic supplementary material

Supplementary material

Supplementary file 1

Supplementary file 2

Supplementary file 3

Supplementary file 4
