# Supplementary material for: Transcriptomic profiling of the human brain reveals that altered synaptic gene expression is associated with chronological aging
Source: Sci Rep. 2017 Dec 4;7:16890. doi: 10.1038/s41598-017-17322-0 (PMC5715102; doi:10.1038/s41598-017-17322-0)
Supplement: Supplementary file 1 — Supplementary material [file 41598_2017_17322_MOESM1_ESM.pdf]

Transcriptomic profiling of the human brain reveals that altered synaptic gene expression is associated with chronological aging

Allissa A. Dillman[1,6], Elisa Majounie[2], Jinhui Ding[3], J Raphael Gibbs[3], Dena Hernandez[4], Sampath Arepalli[4], Bryan J Traynor[5], Andrew B. Singleton[2], Dagmar Galter[6], Mark R. Cookson[1,*]

Supplementary material:

Supplementary Figures 1-11
Supplementary Files 1-4

Supplementary figures

Supplementary figure 1. Samples used with biological and methodological covariates analyzed. (a) Clustering dendrogram based on Euclidean distance (vertical scale) for the samples used in this study. (b) Heatmap of the major variables tested in the WGCNA model, with colors indicated by the scale below the figure. For Gender, black indicates male, white indicates female. RIN, RNA integrity number; PMI, post- mortem interval.

Supplementary figure 2. WGCNA network approximates a scale-free topology. Various values of the soft thresholding power (horizontal axes) were tested for their relationship to scale free topology model fit (A) and the mean connectivity for all genes in the network (B). Based on these, we chose a soft threshold power of 6, which had a correlation to a scale-free topology of R^2~0.9.

Supplementary figure 3. Correlations of eigengenes of network modules with covariates. The module eigengenes (ME) for the constructed WGCNA network modules on the left hand side of the chart were correlated against technical and biological variables along the lower horizontal side of the chart (RIN, RNA integrity number; PMI, post-mortem interval). Each box lists the correlation coefficient and associated p value in parenthesis and boxes are colored by strength of correlation from -1 (blue) to 1 (red).

Supplementary figure 4. Additional modules showing age associations. (a-c) Royal blue module that includes GO terms related to regulatory processes (a), visualized with the most highly expressed transcript per gene (b; circles are sized by inverse R with aging, edges by strength of coexpression). As an example gene, the single

TGFB1 transcript showed negative correlation with age (c). (d-f) Purple that includes GO terms related to ER stress (d), visualized with the most highly expressed transcript per gene (e; circles are sized by inverse R with aging, edges by strength of coexpression). As an example gene, multiple ATF6 transcripts showed negative correlation with age (f).

Supplementary figure 5. Validation of age associations against microarray datasets. Estimates of the correlation of genes with age in this study (horizontal axes) compared to estimates from our prior array study (a) or an independent microarray study (b). Circles are sized by the –log10(p) for the current study and colored by the WGCNA module.

Supplementary figure 6. Correlations of age associations across brain regions in the GTEx dataset. For each region in the GTEx dataset as listed above and to the right of the figure, the overall correlations of associations with age are shown in panels above the diagonal, the distributions of association values on the diagonal and pairwise plots for all genes below the diagonal.

Supplementary figure 7. Markers of principle cell types and aging in the GTEx dataset. The vertical axes show that association of gene expression with age in an unbiased set of markers of principal cell types of the brain along the horizontal axes in the frontal cortex (a) or cerebellum (b). Each point is sized by the –log10(p) for association and colored by the RNA-Seq module assigned for each brain region separately.

Supplementary figure 8. PSEA estimates of the contribution of changes in cellularity to observed gene expression profiles in the aging brain. (a) Component plus residual plots for the human RNA-Seq dataset generated in this study against estimated signal for (from left to right) astrocytes, microglia, neurons and oligodendrocytes on the horizontal axes against components plus residuals of expression on the y axes for the marker genes PPP1R3C, HLA-DRA, KCNK1 and DAAM2 (from top to bottom). Each marker gene shows, as expected, a highly significant positive correlation with proportion of cells estimated in each sample using PSEA. (b) Correlations of age of sample with estimates of markers of (from top to bottom) astrocytes, microglia, neurons and oligodendrocytes. Note that in this dataset there is a negative correlation between neuronal markers and age whereas the opposite is true for estimates of oligodendrocytes. (c) Component plus residuals plots for genes that show high correlations with age, (from top to bottom) TMEM196, ADRA2C, RHBDL3 and HSF4, against estimated signal for (from left to right) astrocytes, microglia, neurons and oligodendrocytes. None of these genes were restricted to any specific cell type, in contrast to the cell specific markers in (a).

Supplementary figure 9. Examples of neuronal markers that show variable associations with aging. SST (a-c) and TMEM130 (d-f) are both predominantly expressed in neurons based on single cell RNA-Seq data (a,d) but show different associations with age in our dataset (b,d, with log2 normalized expression values on equivalent vertical axes for comparison). PSEA in our data further confirms that SST is neuronally enriched and TMEM130 specific for neurons.

Supplementary figure 10. Age associations with and without correction for neuronal estimates in PSEA in the GTEx dataset. We examined a series of genes that were negatively correlated with age in (from left to right) the caudate, cerebellum, dorsolateral prefrontal cortex, frontal cortex and putamen. For each gene, the horizontal axis shows the uncorrected correlation between expression and age and the vertical axis shows the residual correlation with age after correction for the estimate of neurons in each sample, based on PSEA. Filled circles indicate those that remain significant (nominal $p<0.05$) after correction and circles are sized by p after correction.

Supplementary figure 11. Improved detection of age associated genes by RNA-Seq. Gene Significance for Age is plotted for (a) all genes, (b) genes in the brown RNASeq module (c) green RNASeq module or (d) tan RNASeq module for our prior array study and for the current RNASeq study. Note that while the overall distribution of values of GS age are similar (a), in modules that show associations with age the distribution is more restricted in the RNASeq data compared to arrays.

Supplementary Files

Supplementary file 1. Samples used and demographics

A table of the sample identifiers (column 1) against methodological variables RIN (RNA integrity number; raw values), PMI (post-mortem interval, hours) and batch of sequencing (numeric factors of date of sequencing) plus the biological variables gender (1=male, 2=female) and age of the donor in years.

Supplementary file 2. Reads and alignments for accepted samples. Columns are sample identifier, number of reads sequenced, total number of bases sequenced, number of aligned reads to the human genome, the proportion of reads aligned to the human genome compare to total reads.

Supplementary file 3. WGCNA modules and age associations. We used WGCNA to assign transcripts from the aligned reads in the frontal cortex to modules labelled with named colors. For each transcript (columns A-B), the file lists; the associated gene symbol and chromosome of the gene (columns C-D); the assigned module color in WGCNA (column E); gene significance (Pearson's R) for the transcript with age and associated p value (columns F-G); and module membership and associated p values for each potential module (columns H-BC). Note that unassigned genes are included in the 'grey' module.

Supplementary file 4. Gene ontology enrichment for WGCNA modules. Each tab details the gene ontology output from gProfileR for each of the named WGCNA modules only significant terms (column A) are listed along with their p value (column B), the number of genes within a term (column C), the number of module genes (column D) and the number of genes overlapping between B and D (overlap

size, column E). Recall (column F) and precision (column G) are the proportions of overlapping genes found in the query or in the term, respectively. The GO term queried, domain, subgraph, term name and relative depth are in columns H-L and the gene names recovered in each term are in column M. For domain; BP=biological process, CC=cellular compartment, cor=CORUM protein complexes, hpa=human protein atlas, keg=Kyoto encyclopedia of genes and genomics, MF= molecular function, mi=mirbase microRNAs, omi=online mendelian inheritance in man, rea=Reactome, tf=TRANSFAC transcription factor database

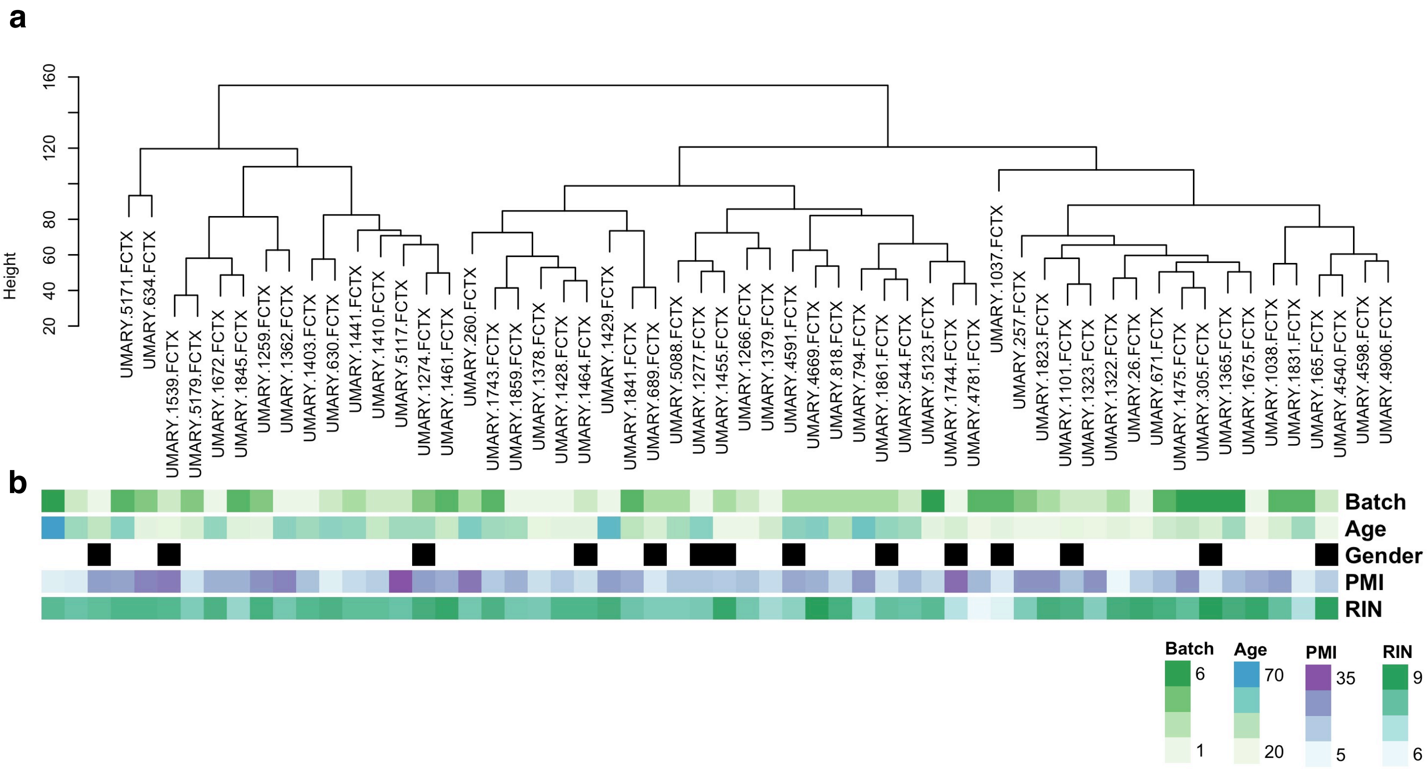

Supplementary figure S1

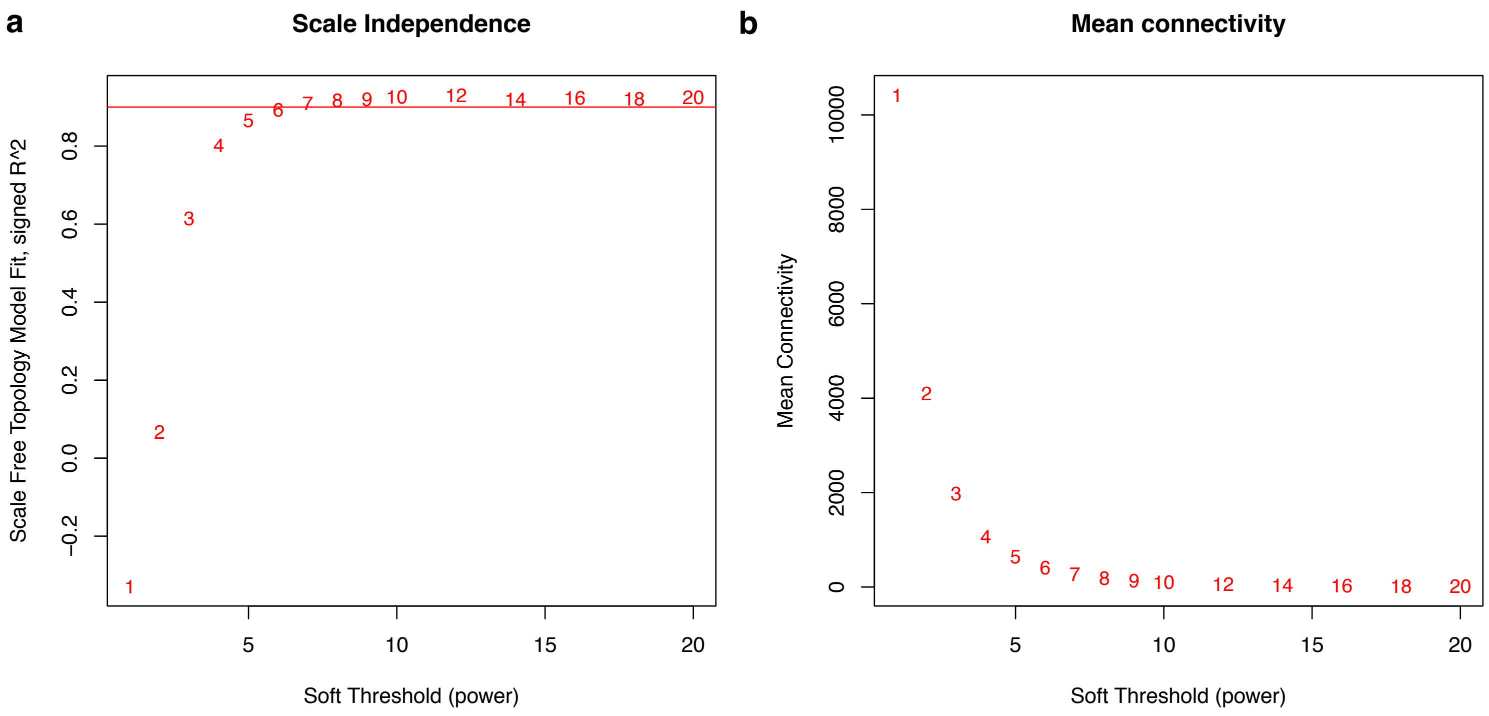

Supplementary figure S2

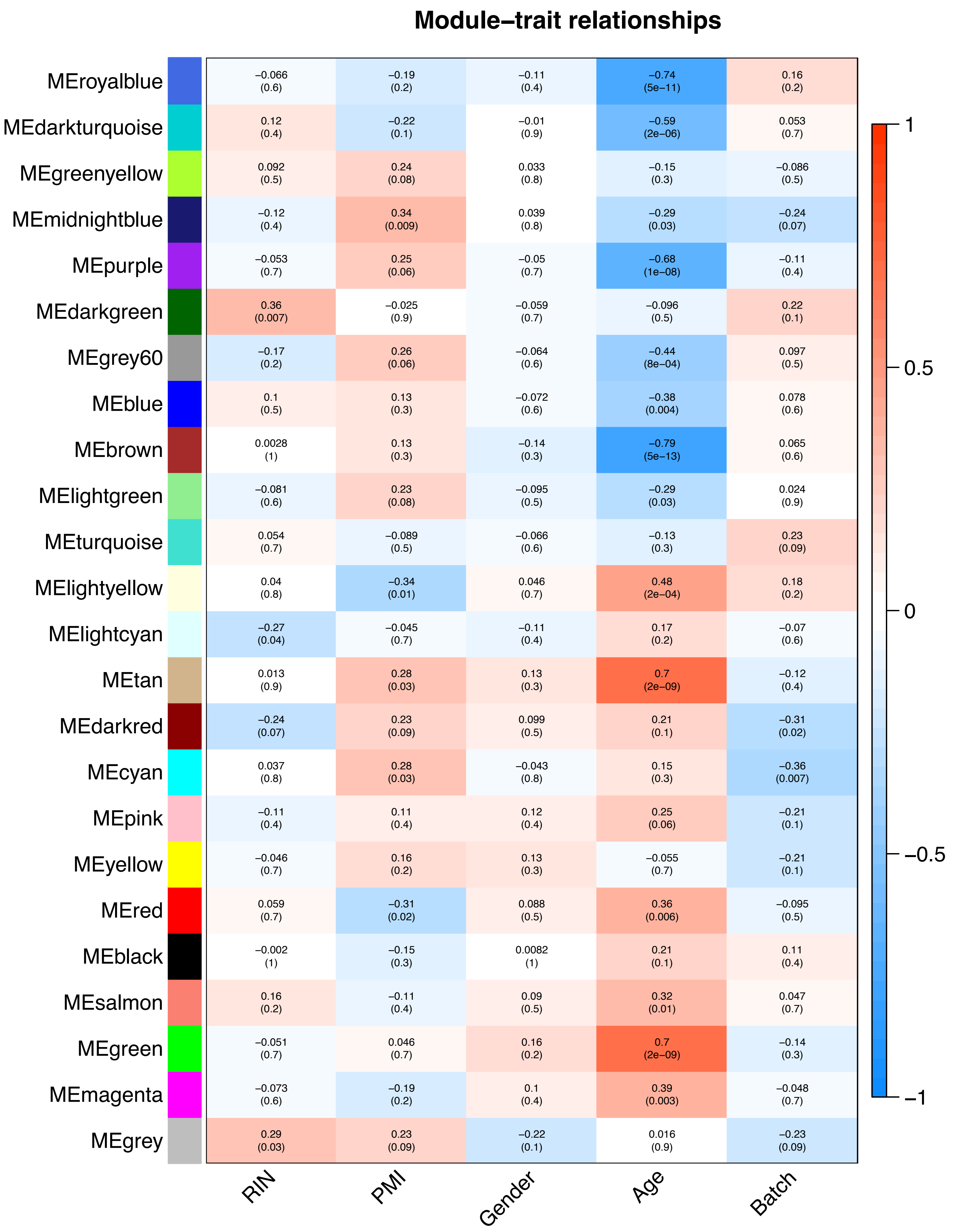

Supplementary figure S3

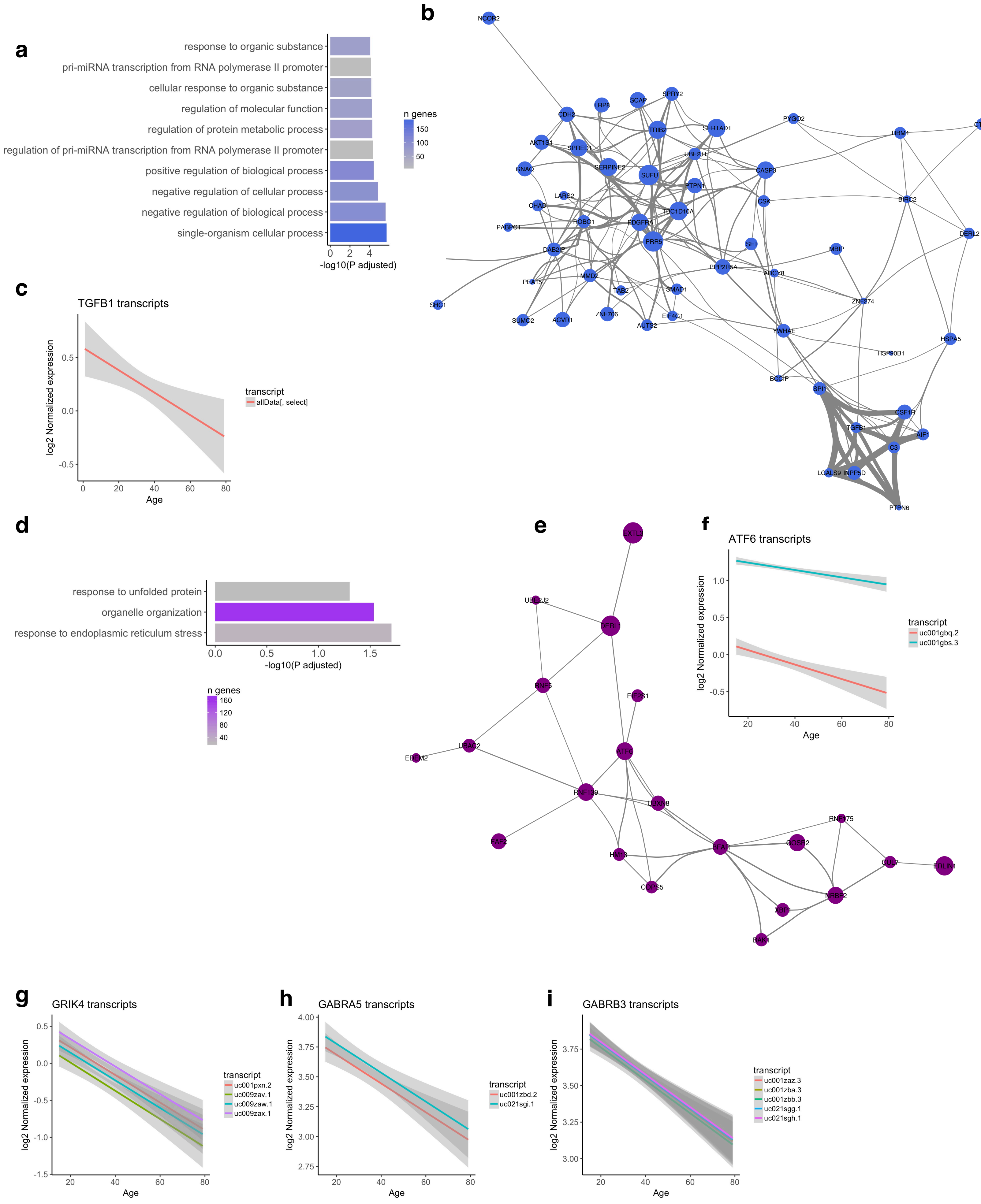

Supplementary figure S4

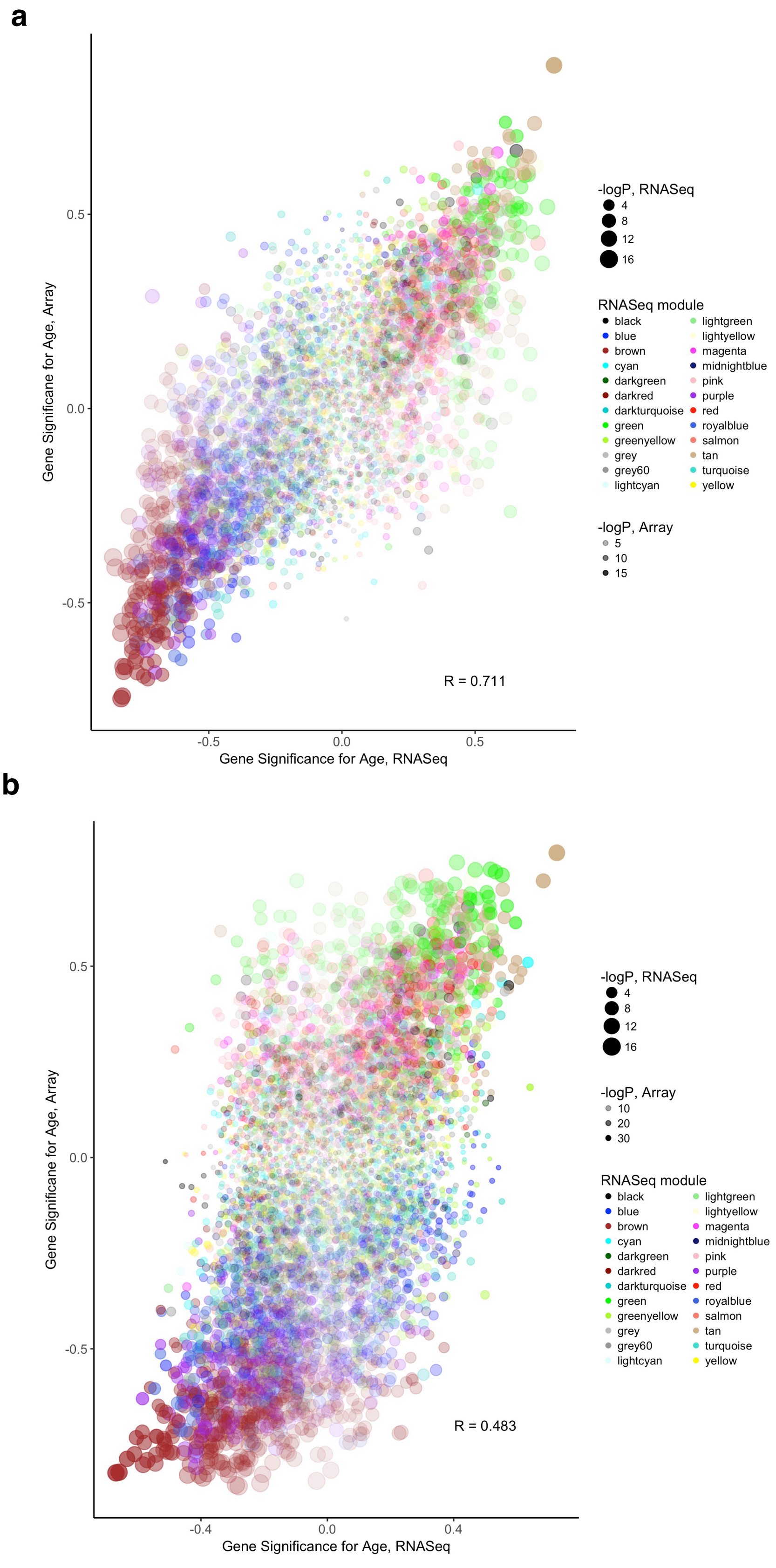

Supplementary figure S5

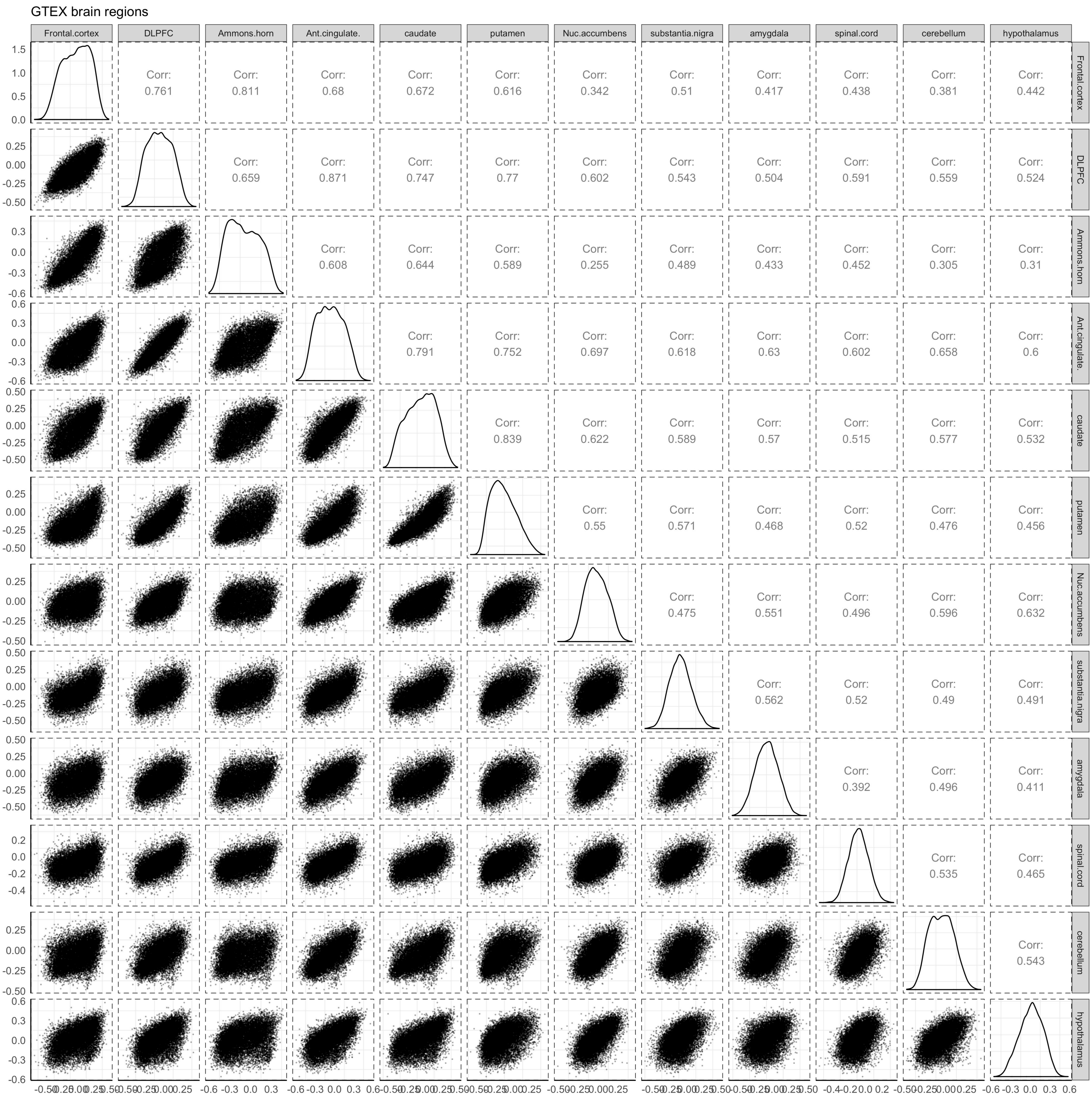

Supplementary figure S6

a

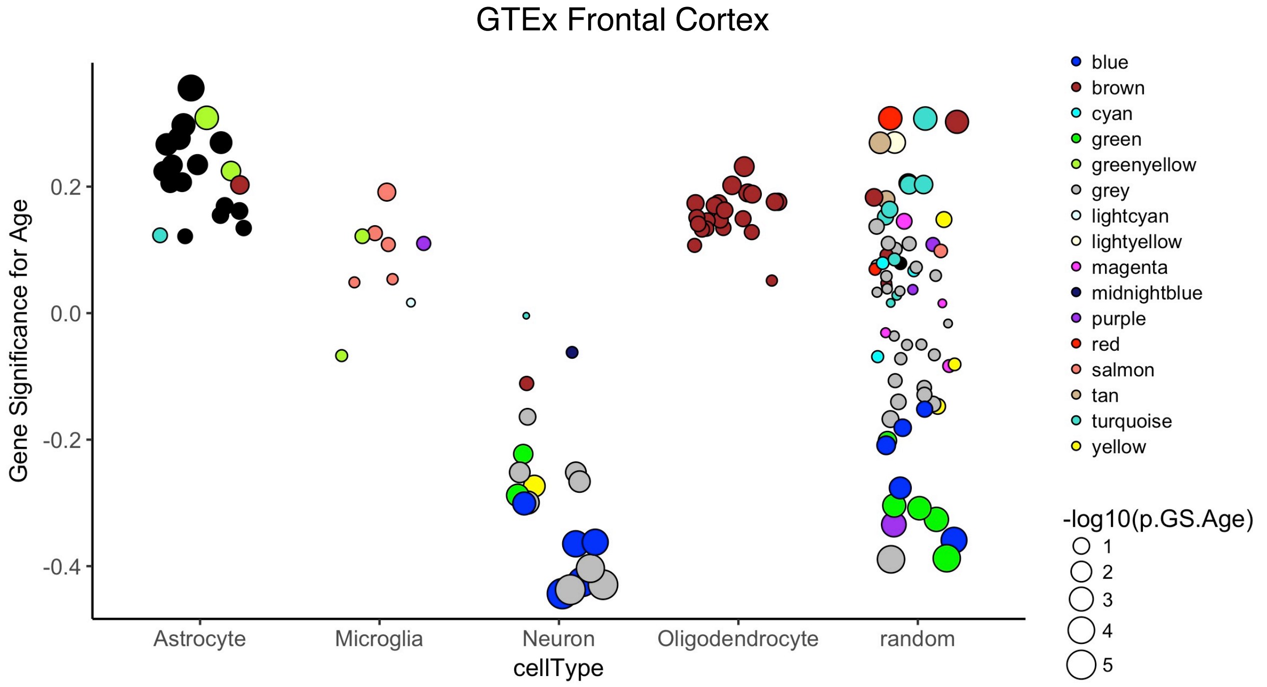

GTEx Frontal Cortex
Gene Significance for Age
0.2
0.0
-0.2
-0.4
Astrocyte
Microglia
Neuron
Oligodendrocyte
random
cellType
blue
brown
cyan
green
greenyellow
grey
lightcyan
lightyellow
magenta
midnightblue
purple
red
salmon
tan
turquoise
yellow
-log10(p.GS.Age)
1
2
3
4
5

b

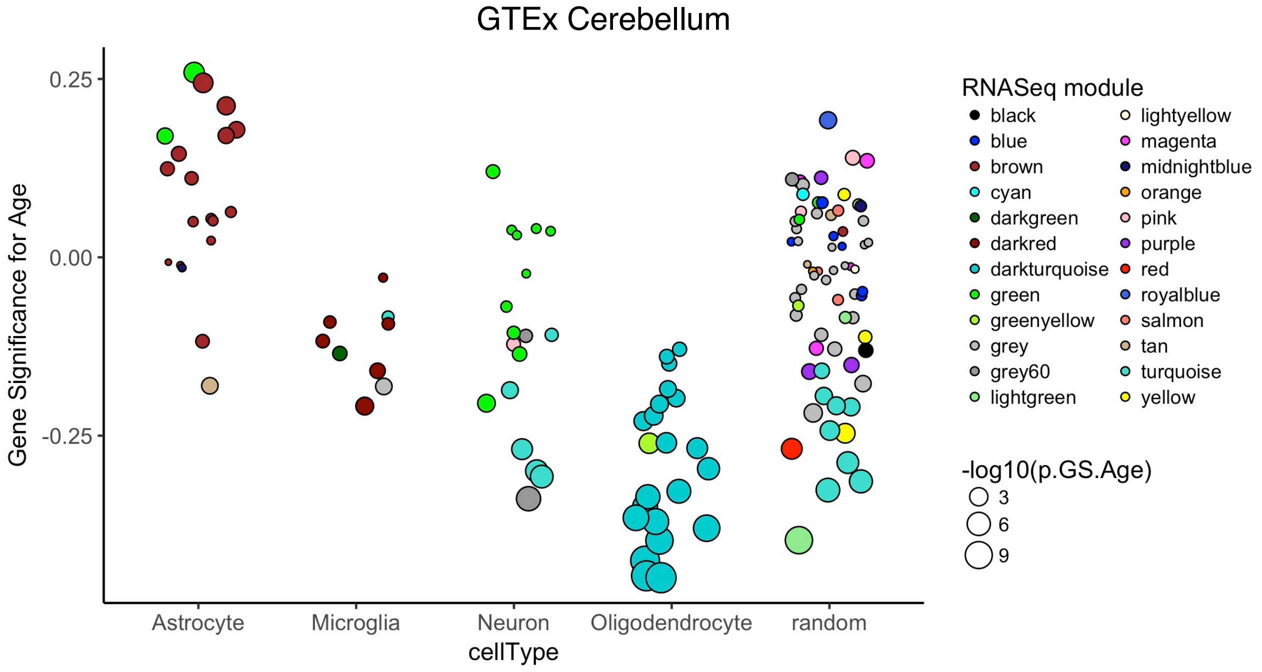

GTEx Cerebellum
Gene Significance for Age
0.25
0.00
-0.25
Astrocyte
Microglia
Neuron
Oligodendrocyte
random
cellType
RNASeq module
black
blue
brown
cyan
darkgreen
darkred
darkturquoise
green
greenyellow
grey
grey60
lightgreen
lightyellow
magenta
midnightblue
orange
pink
purple
red
royalblue
salmon
tan
turquoise
yellow
-log10(p.GS.Age)
3
6
9

Figure S7

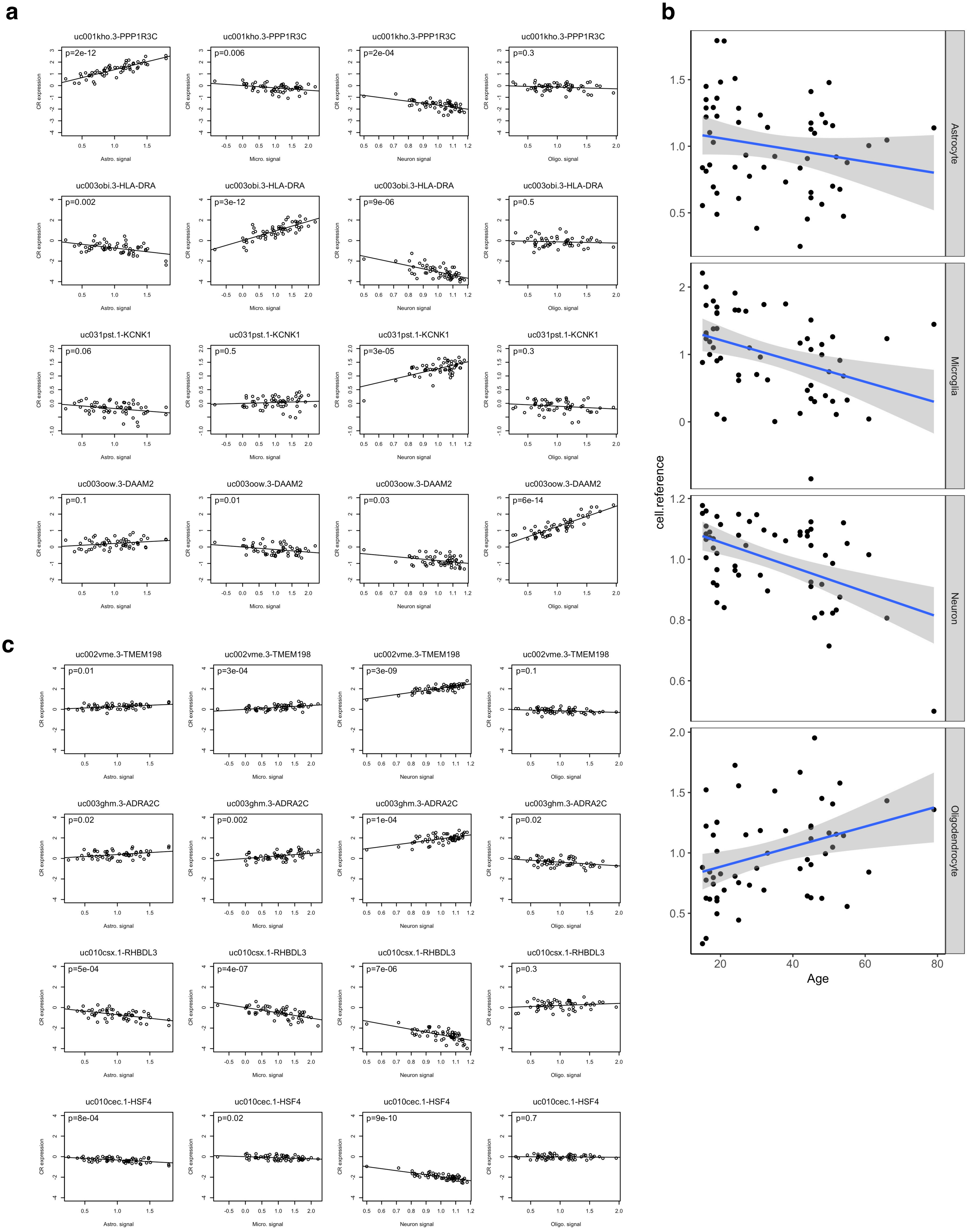
a
uc001kho.3-PPP1R3C
p=2e-12
p=0.006
p=2e-04
p=0.3
uc003obi.3-HLA-DRA
p=0.002
p=3e-12
p=9e-06
p=0.5
uc031pst.1-KCNK1
p=0.06
p=0.5
p=3e-05
p=0.3
uc003oow.3-DAAM2
p=0.1
p=0.01
p=0.03
p=6e-14
CR expression
Astro. signal
Micro. signal
Neuron signal
Oligo. signal
b
Astrocyte
Microglia
Neuron
Oligodendrocyte
cell.reference
Age
c
uc002vme.3-TMEM198
p=0.01
p=3e-04
p=3e-09
p=0.1
uc003ghm.3-ADRA2C
p=0.02
p=0.002
p=1e-04
p=0.02
uc010csx.1-RHBDL3
p=5e-04
p=4e-07
p=7e-06
p=0.3
uc010cec.1-HSF4
p=8e-04
p=0.02
p=9e-10
p=0.7

Figure S8

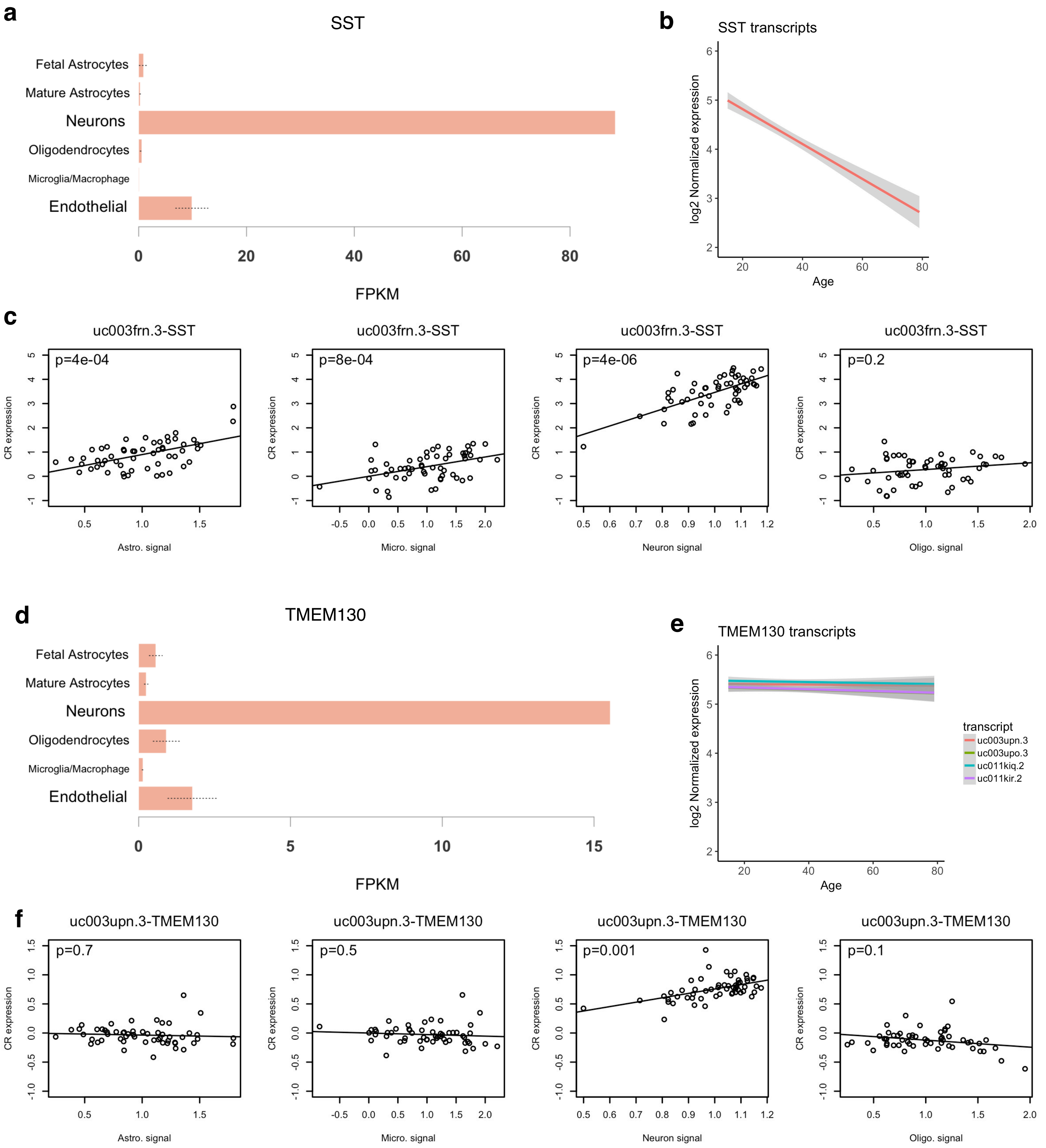
a
SST
Fetal Astrocytes
Mature Astrocytes
Neurons
Oligodendrocytes
Microglia/Macrophage
Endothelial
0
20
40
60
80
FPKM
b
SST transcripts
log2 Normalized expression
Age
c
uc003frn.3-SST
p=4e-04
p=8e-04
p=4e-06
p=0.2
CR expression
Astro. signal
Micro. signal
Neuron signal
Oligo. signal
d
TMEM130
Fetal Astrocytes
Mature Astrocytes
Neurons
Oligodendrocytes
Microglia/Macrophage
Endothelial
0
5
10
15
FPKM
e
TMEM130 transcripts
log2 Normalized expression
Age
transcript
uc003upn.3
uc003upo.3
uc011kiq.2
uc011kir.2
f
uc003upn.3-TMEM130
p=0.7
p=0.5
p=0.001
p=0.1
CR expression
Astro. signal
Micro. signal
Neuron signal
Oligo. signal

Figure S9

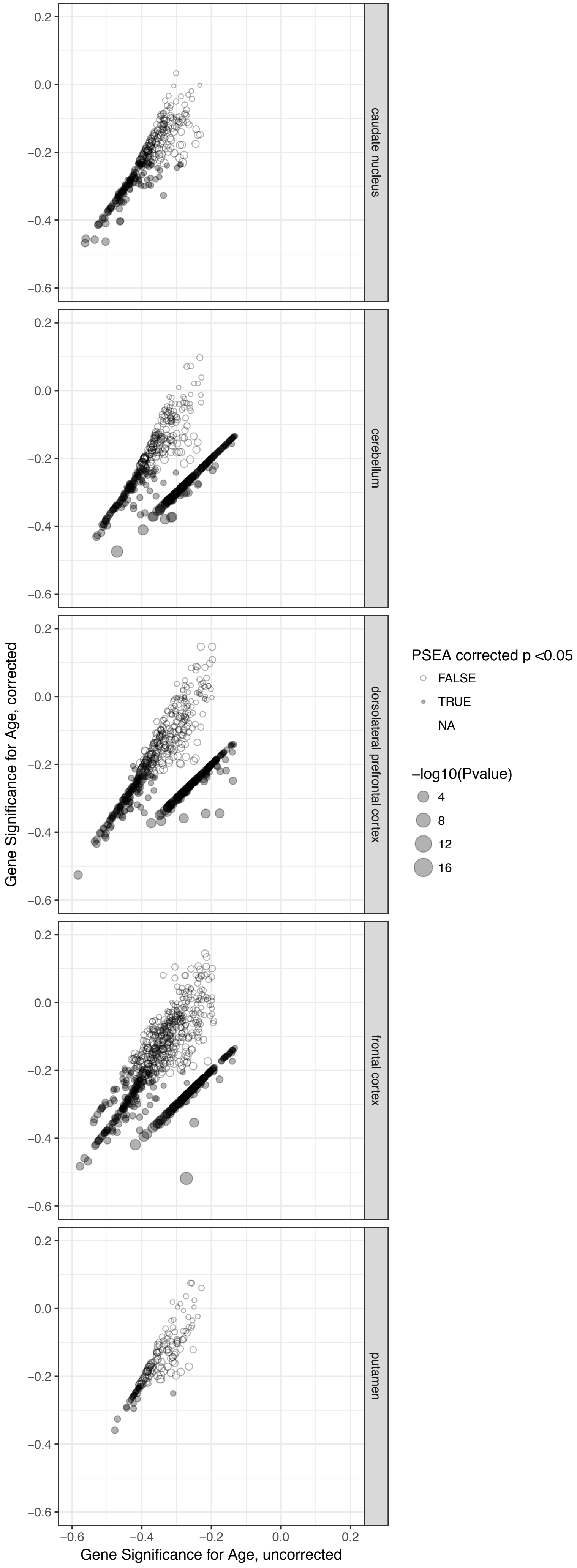

caudate nucleus
cerebellum
dorsolateral prefrontal cortex
frontal cortex
putamen
Gene Significance for Age, corrected
Gene Significance for Age, uncorrected
PSEA corrected p <0.05
FALSE
TRUE
NA
−log10(Pvalue)
4
8
12
16

Figure S10

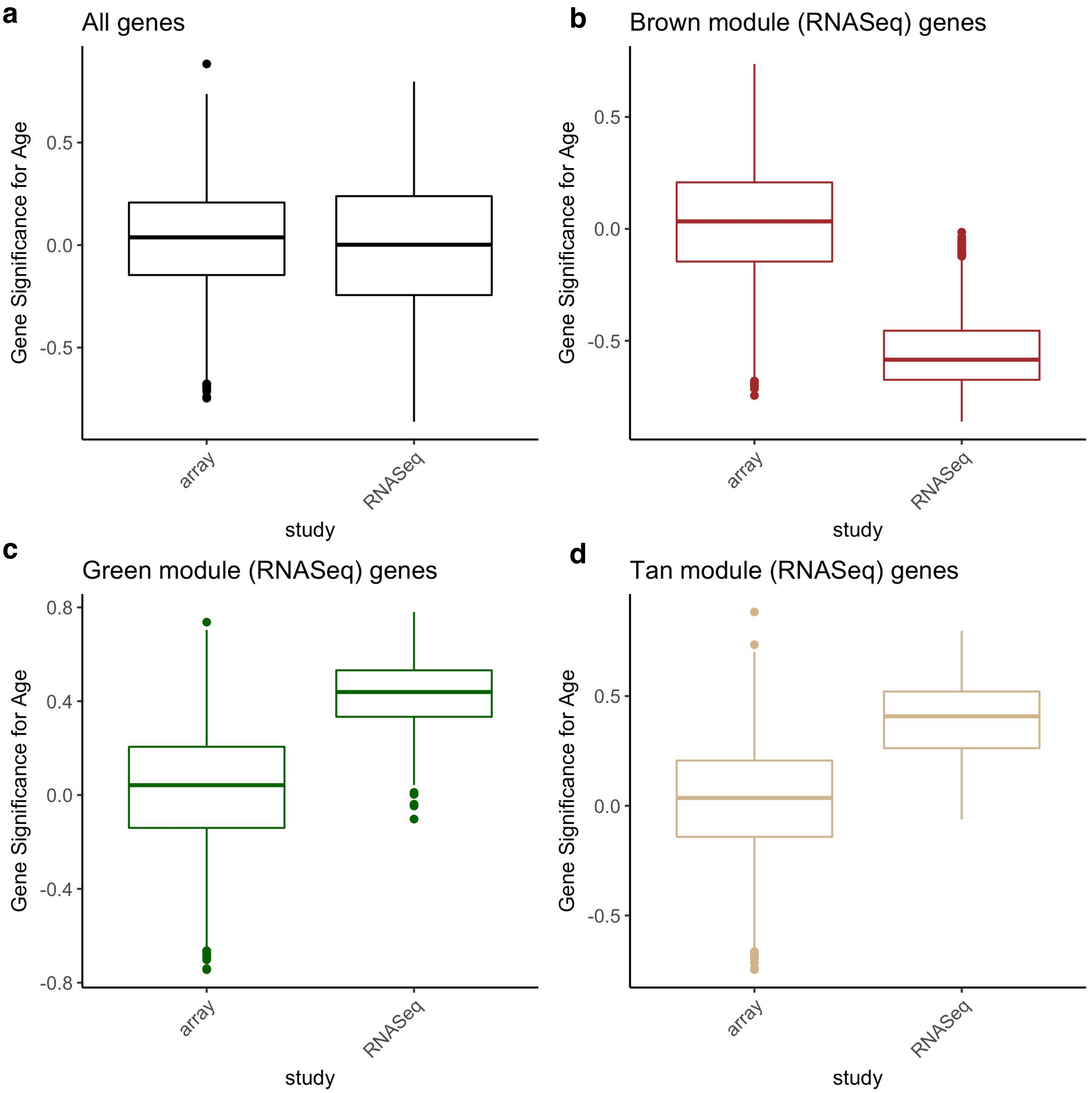
a
All genes
b
Brown module (RNASeq) genes
c
Green module (RNASeq) genes
d
Tan module (RNASeq) genes
Gene Significance for Age
study
array
RNASeq
0.5
0.0
-0.5
0.8
0.4
-0.4
-0.8

Figure S11
